# Kastor: a reference-based comparative approach for assessment and correction of gene-fragmenting errors in long-read assemblies of small genomes

**DOI:** 10.1186/s12864-025-11569-y

**Published:** 2025-04-18

**Authors:** Janet S.H. Lorv, Brendan J. McConkey

**Affiliations:** Department of Biology, University of Waterloo, Waterloo, ON Canada

**Keywords:** Long read sequencing, Nanopore sequencing, Genome assembly, Error correction, Assembly errors, Sequence errors, Oxford nanopore technologies, MinION sequencing

## Abstract

Long read sequencing technologies provide an efficient approach to generating highly contiguous and informative assemblies. However, higher relative error rates can introduce frameshifts and premature stop codons that pseudogenize genes, hindering downstream analyses. We developed a software tool that detects gene-fragmenting errors in draft assemblies of small genomes through comparison with a curated set of reference genome sequences and raw read information. In our presented example, detected errors represent less than 0.05% of the genome, but when corrected reduced the rate of pseudogenes from 23.3 to 5.6% in example long read assemblies, comparable to the rate of pseudogenes in short read assemblies. We demonstrate that this software can detect assembly errors in long read assemblies generated from small genomes and correct them to de-fragment genes.

**Supplementary Information:**

The online version contains supplementary material available at 10.1186/s12864-025-11569-y.

## Background

The number of microbial and eukaryotic genome sequences have increased exponentially in response to decreasing costs and maturation of sequencing technologies. The increased availability of sequencing has been invaluable to the research community and has expanded to include uses such as tracking of bacterial outbreaks [1] and providing insight into micro-evolution processes [2]. However, sequencing technologies are not without challenges [3]. Historically, the most common sequencing technology, Illumina, provides high yield and high quality (< 1% error rate) short paired-end reads ranging from 75 to 300 bp [4]. Although highly accurate, assemblies generated using solely these short fragments may suffer from gaps in coverage and poorly resolved repetitive regions leading to difficulties in ordering and joining of sequences flanking these regions. Consequently, these assemblies often have poor contiguity and resolve into numerous small contigs [5]. This often results in fragmented draft genomes that can hinder downstream studies, such as those involving repetitive sequences, structural rearrangements, transpositions and insertion sequences [2, 6, 7].

In part to alleviate these concerns, long read sequencing technologies such as PacBio’s single molecule read-time (SMRT) and Oxford Nanopore Technology’s nanopore sequencers, have been developed and continue to be refined. Depending on the long-read sequencing platform, library preparation, and sequencing chemistry used, generated reads can on average be greater than 10 kilobases and in some cases up to 2 M base pairs [8, 9]. These longer reads can encompass full length repeat regions allowing for resolution of both complete circular bacterial genomes [8, 10] and larger eukaryotic chromosomes [11].

One disadvantage to long read sequencing is that the current methodology is typically more error-prone than their short-read counterparts [7, 12, 13]. PacBio’s SMRT sequencing initially had a single pass error rate of ~ 13% (RSII with P6-C4 chemistry), but this error rate has since been reduced to < 1% using circular consensus sequencing (CCS) [14]. This improvement utilizes higher sequencing of sub-reads and calls a sequence consensus per read, compensating for random errors in each individual sequencing pass of the read. Similar to PacBio, ONT’s MinION sequencer had a similar initial error rate, where 1D reads sequenced on a minION R9.4 flowcell had an average error rate of up to 15% [15]. Continuous improvements over the years to both flowcell technology, sequencing chemistry, and more accurate basecalling models has further reduced the read error rate to approximately 1% [16] with a recent v14 chemistry kit released in late 2022 (R10.4,1) using the slower, but superior basecalling model (SUP basecalling) [17]. Improvements to read accuracy were attributed in part to more accurate basecalling of homopolymers. Better accuracy in basecalling has also been improved through sequencing of duplex reads, where sequencing of both DNA strands allows for direct alignments of signal data in complementary strands, improving estimates of homopolymer length. In one study, a subsample of 1000 duplex reads was demonstrated to have a modal accuracy of 99.9% when mapped back to the reference [16].

Despite improvements in sequencing quality for both systems, effective sequence correction can be beneficial [11, 18]. Errors in long read sequences are predominantly short insertions and deletions associated with homopolymers [12, 13, 19]. In fact, homopolymer indels account for 92% of errors in PacBio CCS reads [14]. For nanopore sequencing, sequence errors were speculated to be potentially inherent to the base-calling method, resulting from k-mer miscalls due to limitations in the precision of basecalling models during signal interpretation, particularly for low complexity regions [18, 19, 20]. In response to these errors in long-read sequencing, error correction algorithms have been developed and can come pre-packaged with assemblers such as wtdbg2 [21] and Canu [22]. However, the most frequent use-case occurs at the last stage of assembly, where complementary read information specific to the target organism is used to identify final errors and polish the assembly, such as Arrow [23] and Nanopolish [10]. Error correction can be accomplished using both long reads and short reads. With long reads, raw reads mapped to draft assembly are used to correct sequence errors via methods such as read consensus [24], HMM identified signal-to-nucleotide differences [10], and neural network model classified errors [25]. One standardized bacterial genomic assembly pipeline consists of long read assembly using popular assembly software Flye and correction using ONT’s neural network-based polisher Medaka [25, 26].

Even with long read based error correction, these assemblies may still fall short in per-base quality compared to Illumina assemblies [27]. Long reads are capable of resolving chromosomes and plastids into single, contiguous sequences however [28], and many researchers are opting for a hybrid approach incorporating both long and short reads [7, 29]. These hybrid assemblies can be generated either using supplemental long reads to direct assembly with high quality short reads [5, 30], or a long-read assembly is assembled [22, 31] then polished using short reads mapped to a draft assembly to identify sequence errors [32]. Initially, challenges with read mapping strategies to the error-prone long read assemblies, especially for low complexity regions, led to limitations in short read polishing [32]. However, with recent error correction strategies, such as accepting all secondary mapping positions to bypass short read mapping limitations (i.e., Polypolish) [33], and use of a FM-index allowing *k*-mer size flexibility during de-Bruijn graph-based correction (i.e., FMLRC2) [34], short read-polishers have made considerable improvements in long read assembly polishing [35]. Both strategies are sufficient in assembling longer and more contiguous genomes with accuracy greater than 99.99% [11].

Unfortunately, an assembly with high sequence quality may still contain errors that can impede accurate prediction of protein coding regions in long-read based assemblies [3]. Indel errors in long-read assemblies can pseudogenize full genes by introducing frameshifts and premature stop codons [11]. This fragmentation can lead to erroneous interpretations in downstream analyses [7]. As an example, an analysis by Watson and Warr [3] revealed that at least 2746 genes contained indel errors in a 99.8% identity hybrid human assembly [36], and even greater amount of indels were found in a PacBio-only (RSII) assembly [37]. Currently there are difficulties in assessing these inaccuracies without additional sequencing of higher quality short reads, since these errors account for a low percentage of the assembly sequence. Furthermore, as the technology develops and newer accurate basecalling models are released, evaluation of sequence errors are becoming increasing difficult [35]. Traditional assembly evaluation measures such as N50, GC content, L90, and percent identity to homologs do not directly assess gene fragmentation. The benchmarking software BUSCO is one of the few tools available to estimate gene completeness of an assembly by detecting the presence and status of universal single orthologs within a target assembly [38]. However, its focus on orthologs and lack of customizability may lead to potential overestimation of assembly completeness.

In this study we present Kastor, an assembly error assessment software tool that uses a comparative genomic approach to detect gene-fragmenting indel errors, and can also function as an assembly polisher. Unlike common assembly polishers, Kastor does not use additional complementary read information, such as signal data or additional short read sequencing, but rather external reference data already available in the public space for error detection. Kastor compares the final assembled genome with a set of locally aligned reference genomes to identify potential assembly errors. The frequency of alignment differences between the reference genomes and the draft assembly is used to identify consistent differences, which are then marked as candidate errors. These candidates are adjusted or removed, conditional on support from raw read data. Although intended to assess and track sequence errors in long read assemblies, when functioning as an assembly polisher Kastor uses the finalized detected errors as targets for correction to produce a higher quality and more complete assembly. We assessed and validated error identification of Kastor by tracking assembly quality improvements resulting from the corrections made to each assembly. Our test data set includes five bacterial assemblies and a *Plasmodium falciparum* long read assembly as a representative small eukaryotic genome [28, 33, 39]. Additionally, one draft Illumina assembly was also included in our test dataset for comparison. Kastor identified potential assembly errors and defragmented genes in all assemblies. In long read assemblies generated using older sequencing chemistries, we detected fewer than 2000 indel errors associated with 7.5% of known single orthologs as assessed by BUSCO. Upon correction, Kastor improved assembly completeness from 92.2 to 99.7% and reduced the number annotated pseudogenes from 23.3 to 5.6%, comparable to the number of pseudogenes in a corresponding Illumina assembly (4.4%). Kastor detected fewer errors in hybrid assemblies and long read assemblies using newer sequencing chemistries, affirming that greater starting sequence quality arising from sequencer improvements had fewer assembly errors. Therefore, with Kastor error assessment, we are able to detect gene-fragmenting errors in long read assemblies using external reference data without additional sequencing. Correction of these detected errors can improve assembly quality similar to other assembly polishers [28].

## Results

### Kastor detects gene-fragmenting errors in all tested draft assemblies reflecting assembly sequence quality

To assess Kastor’s gene detection capabilities, we ran the software on six small draft genome assemblies, including five prokaryotic genomes and one eukaryotic genome. The test data sets (Supplementary table S1) use different common approaches in genome assembly and represent a range of sequence qualities. Representing earlier iterations of nanopore sequencing, we generated two long read draft assemblies for *Pseudomonas syringae* 508 (Pss508) and *Pseudomonas syringae* TLP2 (PsyTLP2). These assemblies serve as long read only data sets without a complementary short read sequencing and is our main test data. As results were highly similar for both long-read genome assemblies, we focus on the results for Pss508, with PsyTLP2 results presented in supplementary material (Supplementary Tables S2-3 and Figures S1-3). Also, due to significant improvements in software since the initial data collection, new draft assemblies were generated using a recent standardized pipeline with re-basecalled reads using dorado [40], assembly using Flye [26], and polishing using Medaka [25]. These new assemblies are indicated as DFM. As an additional comparative long read only data set, we included a *Mycobacterium tuberculosis* (Mtb) assembly generated as the representative sample incorporating recent improvements in nanopore sequencing chemistry and quality (Q20+). We also included *Citrobacter koseri* (CkMINF_9D) [33] as a hybrid draft assembly sample to represent the common pipeline for microbial genome assembly with improved draft sequence quality due to incorporation of short read data. A published single short-read draft assembly for *Pseudomonas syringae*, Psy642 (Genbank Accession: ADGB00000000), was also included to represent a short read only draft assembly. Lastly, a *Plasmodium falciparum* (PfW2) long read only draft assembly, previously reported as highly error-prone, was included as a representative small eukaryote sample [39].

For each draft assembly, we curated reference genome set(s) targeting different taxonomic levels, ranging from strain (monophyletic subgroups in species complexes) to genus (Supplementary File S4). The primary reference set for each target assembly contains 6 to 15 reference genomes that were curated to avoid groups of highly similar genomes. For example, in the Pss508 reference set, we curated a set of 11 closely related *Pseudomonas syringae* genomes at the species level. These genomes share 94 to 98% pairwise gapped identity between each other, striking a balance between genetic closeness and sequence variability between all genomes including the target assembly (Table S5). An exception is the reference set used for *Plasmodium falciparum* as multiple complete reference genomes were unavailable. In this case, 5 of the best scaffolded genomes from two different sources were chosen to supplement the singular complete genome. Using the default user-specified error threshold of 0.99, Kastor requires all aligned reference genomes to report the same alignment difference within the search window to locate a candidate error (See Methods: Software implementation). Using this comparative genomics approach to error detection, Kastor was able to run each data set in 30 min or less, with most bacterial assemblies completing in under 6 min (Table S6). Longer run-times were indicative of a greater number of error candidates to consider. Run time was approximately proportional to the number of candidate errors, and memory use scaled linearly with genome size and reference set size. With 10 references or fewer, Kastor typically uses ~ 1 Gb of memory per 1 Mb of draft sequence. Kastor’s largest memory consumption was 19.03Gb during testing of PfW2, the largest draft assembly in our test set (21.7 Mb). Due to memory requirements, Kastor is best suited to small genome assemblies. In addition to the above data for Kastor, runtimes and peak memory usage for data extraction from reference genomes using LASTZ are included in supplementary file S4.

Kastor detected less than 2000 indel errors in each bacterial genome, and ~ 40,000 indels in the eukaryote assembly PfW2 without need of any additional short read sequencing (Table 1).

**Table 1:** Number and types of errors detected by Kastor

Organism	Reference set^1^	Detected Errors			
		*Total*	*Insertions*	*Deletions*	*Substitution* ^*2*^
***Pseudomonas syringae*** **508**	Set 1	2900	900	1708	900
	Set 2	657	36	263	358
***Pseudomonas syringae*** **508 (DFM)** ^**3**^	Set 1	1674	187	559	928
	Set 2	1066	28	98	940
***Pseudomonas syringae*** **642**	Set 1	195	71	124	-
***Citrobacter koseri*** **MINF_9D**	Genus	951	225	209	517
	Species	97	9	75	13
***Mycobacterium tuberculosis***	Species	18	3	3	12
***Plasmodium falciparum*** **W2**	Species	40,681	20	40,661	-

1 – Reference data set used for Kastor correction depending on taxonomic closeness to the target assembly (i.e., reference genomes from *Citrobacter* species were included at the genus level, but only *Citrobacter koseri* genomes at the species level. See Supplementary File S4 for Reference set composition

2 – Substitutions were excluded when raw reads were unavailable

3 – basecalled using Dorado and assembled using Flye and Medaka (see Methods)

Overall, Kastor detected errors representing less than 0.035% of the bacterial assemblies and less than 0.175% of the eukaryotic assembly. As expected, Kastor detected fewer errors in draft assemblies with higher initial sequence quality (CkMINF_9D, Psy642, and Mtb). Use of short read sequence quality the Trycycler hybrid assembly, CkMINF_9D, resulted in less than 100 detected errors, similar to the Illumina draft assembly with less than 200 detected errors (Psy642). Most surprisingly, significant improvements in nanopore sequencing read quality are reflected in Kastor detecting only 6 indel errors in the Q20 + long read only Mtb assembly.

In order to determine the functional importance of the putative errors, corrections were applied to detected errors and improvements to assembly completeness were assessed by evaluating the presence of known single universal orthologs in each assembly before and after error correction using BUSCO (Table 2) [38]. Since Kastor can function as an assembly polisher, for comparative purposes we also ran short read assembly polishers, polypolish and FMLRC2, using the provided short read datasets and default parameters on the CkMINF_9D assembly. In all pre-corrected long-read draft assemblies, we confirmed that despite at least one round of assembly polishing with Medaka, the draft assemblies were partially incomplete and included fragmented genes. Here, 39–69 (4.9–8.9%) of Pseudomonadales orthologs were considered fragmented in Pss508 and 22–41 (2.8–5.2%) were missing. On manual inspection we were able to confirm that at least 41 of the 69 fragmented orthologs in Pss508 contained indels resulting in out-of-frame errors. In the hybrid assembly, CkMINF_9D, the use of short reads during assembly improved draft quality, however 3 (0.6%) and 5 (1%) enterobacterales orthologs were still found to be fragmented and missing respectively. The PfW2 assembly was the most error prone with 474 (13.0%) fragmented and 632 (17.4%) missing orthologs. Conversely, the two highest quality draft assemblies (Psy642 and Mtb), had the fewest combined fragmented and missing orthologs with less than five in each case.

**Table 2:** BUSCO assessment of assembly completeness pre- and post-corrected assemblies. Test datasets with available illumina data were corrected using short read Polishers, polypolish and FMLRC, for comparative purposes. Single universal orthologs were determined to be complete, duplicated, fragmented or missing as an evaluation of assembly completeness using BUSCO

Organism	Assembly Type			Correction	Reference set^1^		Status of universal single orthologs^2^			
						*Total*	*Complete*	*Duplicated*	*Fragmented*	*Missing*
***Pseudomonas syringae*** **508**	Draft			-	-	782	672	0	69	41
	Corrected			Kastor	Set 1	782	778	0	4	1
	Corrected			Kastor x2	Set 2	782	780	0	2	1
***Pseudomonas syringae*** **508 (DFM)**	Draft			-	-	782	721	0	39	22
	Corrected			Kastor	Set 1	782	778	0	3	1
	Corrected			Kastor x2	Set 2	782	780	0	1	1
***Pseudomonas syringae*** **642**	Draft			-	-	782	777	0	4	1
	Corrected			Kastor	Set 1	782	777	0	4	1
***Citrobacter koseri*** **MINF_9D** **(hybrid)**	Draft			-	-	440	431	1	3	5
	Corrected			Kastor	Genus	440	434	1	0	5
	Corrected			Kastor	Species	440	435	1	0	4
	Corrected			Polypolish	-	440	434	1	0	5
	Corrected			FMLRC	-	440	434	1	0	5
***Mycobacterium tuberculosis***	Draft			-	-	124	115	6	1	2
	Corrected			Kastor	Species	124	115	6	2	1
	Reference			-	-	124	120	2	1	1
***Plasmodium falciparum*** **W2**	Draft			-	-	3642	2536	0	474	632
	Corrected			Kastor	Species	3642	3487	2	53	100
	Reference					3642	3585	0	3	54

1 – Reference data set used for Kastor correction depending on taxonomic closeness to the target assembly (i.e., reference genomes from *Citrobacter* species were included at the genus level, but only *Citrobacter koseri* genomes at the species level). See methods for details

2 – Status of known single universal orthologs in each corresponding BUSCO lineage dataset. Each dataset was evaluated based on the closest known lineage: *Pseudomonas syringae* with Pseudomonadales; *Citrobacter koseri* and *Enterobacter kobei* with Enterobacterales; *Mycobacterium tuberculosis* with bacteria; and *Plasmodium falciparum* with Plasmodium

Using BUSCO analysis, our correction of Kastor detected errors led to improvements in assembly completeness for long read only and the hybrid assembly, In these cases, we were able to recover previously fragmented and missing orthologs comparable to a respective reference genome. There was an increase to 99.7% completeness in both *Pseudomonas syringae* assemblies with only a single fragmented and a single missing ortholog, comparable to an Illumina draft equivalent (Psy642). For CkMINF_9D, correction of ~ 100 detected errors made slight improvements in assembly completeness with defragmentation of 3 genes. The smaller gains are indicative of the higher sequence quality with a hybrid assembly approach, but gene-fragmenting errors associated with these orthologs were still detectable with Kastor. When compared to the two other short read polishers, Kastor was also able to polish the assemblies to a similar level without use of additional short read sequencing. Of the two remaining bacterial assemblies, Psy642 and Mtb, there was only minimal change, indicative of the already high sequence quality of these assemblies. Fragmented orthologs remaining in Psy642 and duplicated orthologs in Mtb were the result of assembly artifacts rather than sequence quality, such as contig gaps and sequence duplication for the Psy642 and Mtb assemblies respectively.

Lastly, the long read eukaryotic assembly, PfW2, demonstrated the greatest improvements with an increase of 953 detectable complete orthologs and an improvement of assembly completeness from 69.6 to 95.8%. In this case, assembly completeness of PfW2 were similar to scaffold level assemblies rather than the complete reference genome. In all cases, Kastor was able to detect gene-fragmenting errors leading to recovery of complete genes in these assemblies through correction.

One caveat to BUSCO assessment is that the program may overestimate genome completion by only considering conserved and core orthologs within the selected ortholog database, neglecting less conserved genes within the genome. For a more comprehensive view of the functional impact of the gene-fragmenting errors found, we investigated the completeness of the whole genome by annotating each bacterial assembly using NCBI’s prokaryotic genome annotation pipeline (PGAP) to locate both complete genes and pseudogenes (i.e., frameshifted and incomplete genes) [41]. PGAP detected higher levels of gene-fragmentation in the pre-correction draft Pss508 assembly, with 1234 observed pseudogenes (23.3% of total genes), compared to BUSCO (14.1% fragmented and missing), suggesting an overestimation of assembly sequence quality and completeness (Tables 2 and 3). Of these, 1101 (89.2%) of the PGAP annotated pseudogenes were considered frameshifted only, suggesting higher levels gene-fragmenting indels when compared to the short-read assembly Psy642, which contained 25 frameshifted pseudogenes. The other bacterial assemblies follow a similar trend. Draft assembly CkMINF_9D has 137 (3.0%) pseudogenized genes compared to BUSCO’s assessed 8 missing or fragmented orthologs (1.8%). Even the Q20 + draft assembly, Mtb, has an additional 16 pseudogenized genes compared to a reference Mtb genome, suggesting assembly completeness based on highly conserved orthologs may somewhat underestimate assembly completeness.

**Table 3:** Impact of assembly error correction on detection of PGAP annotated fragmented genes on bacterial test datasets

Organism	Assembly Type		Correction		Reference set^1^	PGAP annotated genes^2^		
						*Complete*	*Frameshifted*	*Incomplete*
***Pseudomonas syringae*** **508**	Draft		-		-	3967	1101	133
	Corrected		Kastor		Set 1	4890 (+ 949 / -20)	190 (+ 8/ -917)	106 (+ 8 / -36)
***Pseudomonas syringae*** **642**	Draft		-		-	4981	25	208
	Corrected		Kastor		Set 1	4982 (+ 9 / -8)	21 (+ 1 / -5)	213 (+ 6 / -2)
***Citrobacter koseri*** **MINF_9D**	Draft		-		-	4422	83	54
	Corrected		Kastor		Genus	4455	57	50
	Corrected		Kastor		Species	4476	36	45
	Corrected		Polypolish		-	4484	27	45
***Mycobacterium tuberculosis***	Draft		-		-	4079	90	112
	Corrected		Kastor		Species	4081	87	112
	Reference		-		-	3972	81	105

1 – Reference data set used for Kastor correction depending on taxonomic closeness to the target assembly (i.e., reference genomes from *Citrobacter* species were included at the genus level, but only *Citrobacter koseri* genomes at the species level)

2 – General types of pseudogenes as annotated by the PGAP software. Genes annotated as incomplete include both incomplete genes and pseudogenes with internal stop codons

After Kastor detected errors were corrected, all bacterial assemblies were reassessed with PGAP. Both *Pseudomonas* long read assemblies showed considerable improvements (Table 3); for example in Pss508, Kastor increased the number of completed genes from 3967 (75.0%) to 4890 (92.7%). Tracking the genes between the pre- and post-correction assemblies reveals that 949 completed genes were added after Kastor correction, and 20 genes were removed. The majority (909) of added genes were previously annotated as fragmented due to frameshifts, while a minority (30) were previously incomplete genes. Unexpectedly, 10 new complete genes were also found. Of the 20 completed genes removed from the pool, 16 were no longer predicted in the assembly while three became frameshifted and one became incomplete. Overall, there is a large decrease in the number of frameshifted and incomplete genes. Eight pseudogenes were reclassified as a different type; five frameshifted pseudogenes became incomplete whereas three incomplete pseudogenes became frameshifted.

Following this trend, when Kastor detected errors were corrected in hybrid assembly, CkMINF_9D, there was a decrease in both frameshifted (83 to 36 genes) and incomplete genes (54 to 45 genes) when a species reference set was used (Table 3). Under the assumption that one error impacted each pseudogenized gene, at least 66 of 84 detected indels (79%) are gene-fragmenting errors suggesting that Kastor error detection is highly targeted to assembly errors. Furthermore, when compared to short read polishers, i.e., polypolish, our conservative approach reaches a similar quality differing by only 9 pseudogenes. Even in the highest quality long read assembly (Q20+), Mtb, identification of 6 indels led to a decrease in 3 pseudogenes supporting that the errors detected by Kastor are assembly errors.

In the Illumina short read assembly Psy642 Kastor identified relatively few potential error sites. Post correction there is a gain of nine complete genes along with a loss of eight complete genes, for a net increase of one gene. There was also a net loss of five frameshifted genes and a net gain of six incomplete genes (Table 3). Of the eight complete genes that were lost, three became pseudogenized and five were no longer predicted. Similar to Pss508, Kastor can identify and defragment frameshifted pseudogenes in the Psy642 short-read assembly. Here, three genes were converted from pseudogenes to complete genes. There is an increase in incomplete pseudogenes, but similar to Pss508, two are new, two are a shift in pseudogene type, and only two represent a pseudogenization of previously complete genes. In both Pss508 and Psy642 assembly correction, there is a very low incidence of fragmentation of complete genes (four and three genes respectively) consistent with a low level of false positive corrections. Furthermore, in both high quality short and long read assemblies, errors found with Kastor did not appear to hinder assembly quality post-correction.

**Fig. 1:**
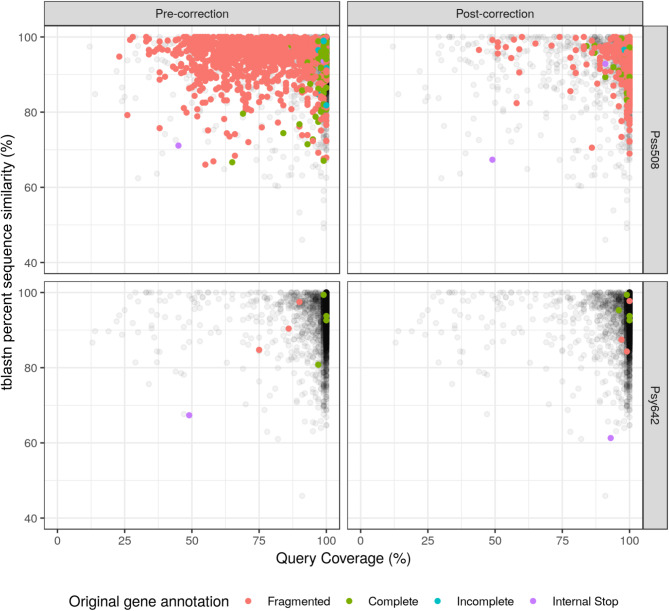
Reduction of fragmentation of identified genes for (**A**) Pss508 long-read assembly, and (**B**) Psy642 short-read Illumina assembly, before and after Kastor correction. RefSeq proteins used in PGAP’s protein homology annotation of pre-correction assemblies were mapped back to each assembly using tblastn and plotted as percent sequence similarity vs. percent sequence coverage. Corrected genes are colored according to the original annotations while non-corrected genes are represented as grey. In detail, fragmented indicate genes where two or more frameshifted gene fragments were found; complete indicates complete genes; incomplete indicate truncated genes at the 5’ or 3’ end; and internal stop indicates genes containing a stop codon truncating the full gene. Gene fragmentation or incompleteness is inferred from lower query coverage when tblastn detects only a fragment of expected gene. Longer and more similar genes were found after correction

To investigate whether Kastor correction has additional impacts beyond de-fragmenting pseudogenes, we assessed the protein product similarity and coverage to the respective RefSeq ortholog used in PGAP’s homology-based annotation [41]. The RefSeq protein orthologs were extracted for each gene with a known product; *de novo* predicted genes were excluded. These RefSeq protein sets were then mapped back to the respective assembly using tblastn and restricted to the best high scoring pair (HSP). We reason that the top HSP would yield the assembly gene equivalent, and fragmented genes should have either poor query coverage or low similarity. We plotted gene query coverage (qcov) to percent sequence similarity for each HSP (Fig. 1).

After error correction, most genes with a correction in both Pss508 and Psy642 showed an average increase in query coverage and similarity regardless of the annotation prior to correction; even previously complete genes showed an improvement (Fig. 1). For Pss508, 1076 genes showed improvements in either query coverage or similarity, with 55.6% of these genes increasing in both scales, 37.4% increasing in query coverage only, and 7.0% increasing in only query similarity. Only 35 genes, representing 3.1% of edited genes, decreased in query coverage and similarity and only 12 of these decreased by greater than 5% for either coverage or similarity. Notably, six of these genes were de-fragmented pseudogenes. Despite being reclassified as complete, these genes had a decrease in similarity with their respective RefSeq orthologs.

Since the majority of genes have no corrections, we compared the general protein product profiles between Pss508 and its sister strain Psy642 (Figure S4). Prior to correction, the Pss508 assembly is highly fragmented compared Psy642, but post correction Pss508 and Psy642 become highly similar in terms of coverage and sequence similarity. This profile similarity suggests that the remaining genes with low query coverage may reflect strain differences rather than assembly errors. However, the post-corrected Pss508 assembly profile still has a greater number of fragmented genes. These non-corrected genes may be false negatives, possibly stemming from insufficient information for error correction. When taken together, our three approaches (BUSCO, PGAP, and gene similarity tracking) indicate that the errors detected with Kastor are highly associated with gene-fragmentation in draft assemblies. More error prone draft assemblies demonstrated higher error rates, which decreases with higher quality draft assemblies. Upon correction of detected errors, Kastor can be used as an assembly polisher, with the Kastor-polished assembly approaching sequence quality levels similar to short read polishers but bypassing the need for additional sequencing data.

### Profile of Kastor detected errors

In general, long read assemblies are expected to be more error prone than short-read assemblies [3]. Many of these errors were previously attributed to poor disambiguation of homopolymer length during assembly [10, 15]. We investigated whether Kastor detected errors in Pss508 follow the same pattern. Unexpectedly, Kastor initially identified a higher level of substitution error candidates than gene-fragmenting indel errors common to long reads with 24,600 and 2000 candidates respectively [12, 13]. However, many of these initial substitution candidates were subsequently dropped as they lacked read support. Only 900 (3.6%) substitutions were retained using a lenient threshold of 10% support, from raw unprocessed reads, of a singular replacement nucleotide. Similarly, indel error candidates have low read support with 561 (28%) of indel error candidates having support using a threshold of 25% of reads. By design, Kastor initially retains all detected indel error candidates. Rather than removing these candidates, the software adjusts the positioning of the error and expected correction. We reason that the systematic association of indel errors with homopolymer repeats in long reads could lead to low levels of read support for the detected indel candidates [3, 15, 19].

After error adjustment, Kastor found a total of 2900 errors in Pss508, accounting for less than 0.05% of the assembly (Table 1). Indel errors make up the majority of errors at 69% of all errors passing thresholds, with the majority of indels being false deletions. Comparatively, a much smaller number of errors were found in Psy642 which is likely reflective of the overall increased accuracy of nucleotide calling in Illumina assemblies [4]. Although a higher number of deletion errors (124) were detected than insertion errors (71), the disparity between the two error types is less pronounced than in the Pss508 assembly.

There is also a difference in the location of indel errors between the two assemblies. Although coding sequences account for 89.1% of the Pss508 genome, only 1317 (67%) of the indels found in Pss508 were within annotated genes while the remaining 683 (32.6%) were in intergenic regions. In contrast, only 17 (~ 9%) of the errors in Psy642 were associated with annotated genes. This may be due to the low rate of indel changes within genes when comparing between bacterial genomes, so detected indels are more likely to represent errors. Conversely, intergenic regions may have more indel associated differences between genomes, making it more difficult to distinguish indel errors from strain-to-strain variation.

When taken together with overall improvement and defragmentation of error containing genes (Fig. 1), the number of indels found in pseudogenes and low number of gene-associated errors in Illumina indicates the corrected within-gene indel errors in the Pss508 assembly are unlikely to be spurious errors. Conversely, there are an elevated number of indel errors found in the intergenic region for both assemblies, despite representing < 11% of the genome being intergenic. When manually inspected, some of these errors were found in variable length homopolymers in otherwise conserved regulatory RNA regions. In non-conserved intergenic regions, high nucleotide variability may lead to poor mapping of the region and consequently high levels of indel differences. The increases of indels may make these regions prone to false detection that may contribute to higher level of intergenic corrections.

**Fig. 2:**
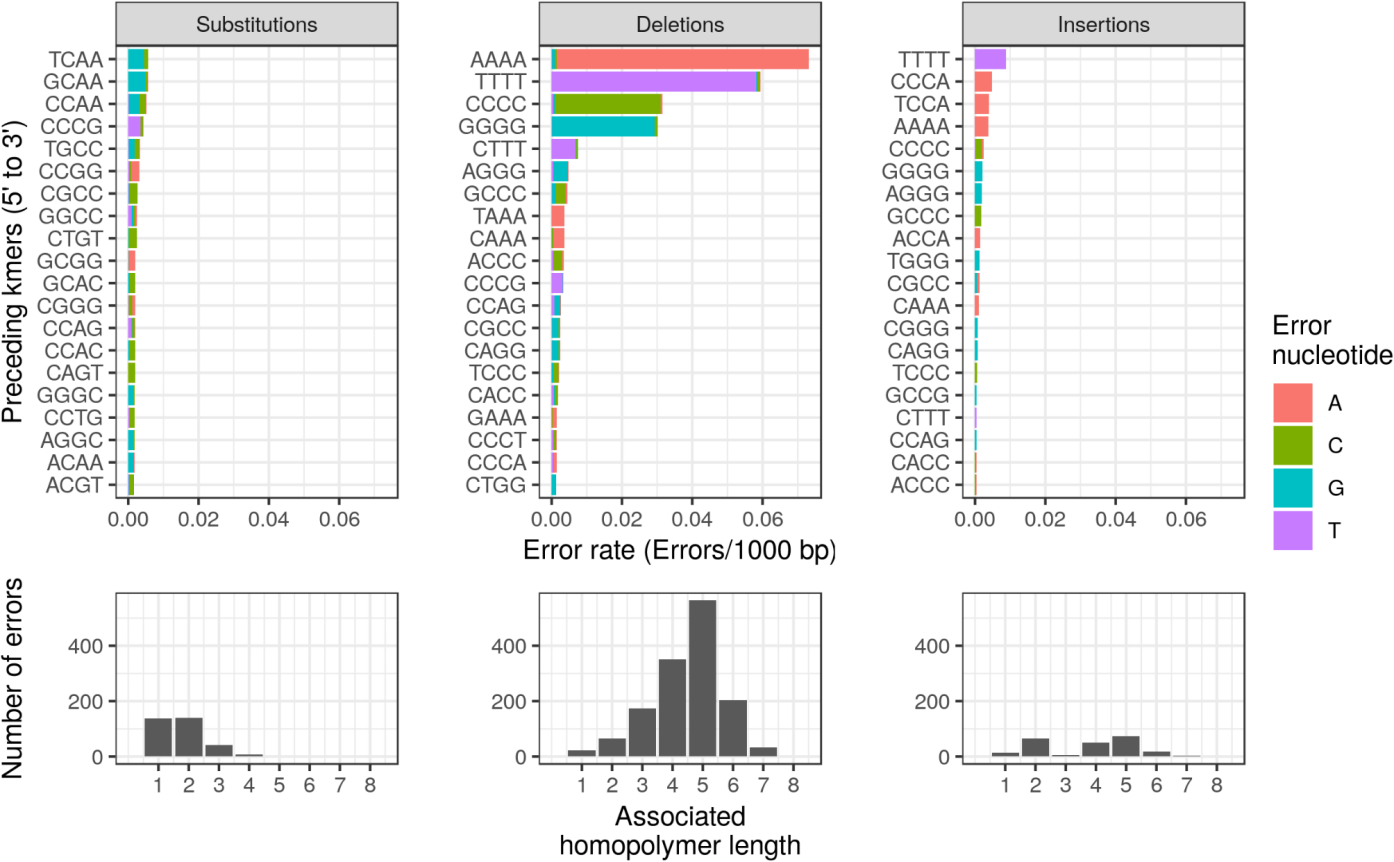
Breakdown of the genomic sequence preceding detected errors in the Pss508 assembly. Top plots: Top 20 k-mers (k = 4) upstream of detected errors separated by error type. Each bar is colored with the associated erroneous nucleotide. Bottom plots: the distribution of the length of upstream homopolymers for the respective error type

Since nanopore sequences are base-called as 5 or 6-mer events for R9.4 flowcells, we investigated whether detected errors are associated with specific k-mers in Pss508 by extracting the upstream 4-mer for each detected error [15]. When the top 20 k-mers were plotted for each error type (Fig. 2), the majority of errors are associated with homopolymers which are expected to be more error-prone [10]. However, the associated homopolymers differ for each error type. In general, substitution errors appear to occur frequently following small homopolymers, length 3 or less, or single nucleotides. There is no dominant 4-mer associated with substitutions suggesting that they are more randomly distributed in the assembly. Furthermore, substitution errors are more likely to be either a guanine or cytosine regardless of the upstream 4-mer.

In contrast, deletion errors are highly associated with homopolymers from two to eight nucleotides long (Fig. 2). Although there is a large range, the greatest number of identified deletion errors occur following four to five stretches of the same nucleotide [19]. Unexpectedly, despite a relatively high GC content of 59%, the most common errors were associated with long stretches of adenine and thymine followed by around half as many errors associated with guanine and cytosine. In all cases, the detected error tends to match the associated homopolymer, but there is a small incidence of errors where a non-repeated nucleotide is deleted. Taken together, the prominence of deletion errors occurring after long stretches of the same nucleotide may reflect difficulties in determining accurate homopolymer length during basecalling. The uncertainty and variability in homopolymer length between reads can lead to more conservative estimates of length and therefore introduce erroneous deletions [15].

**Fig. 3:**
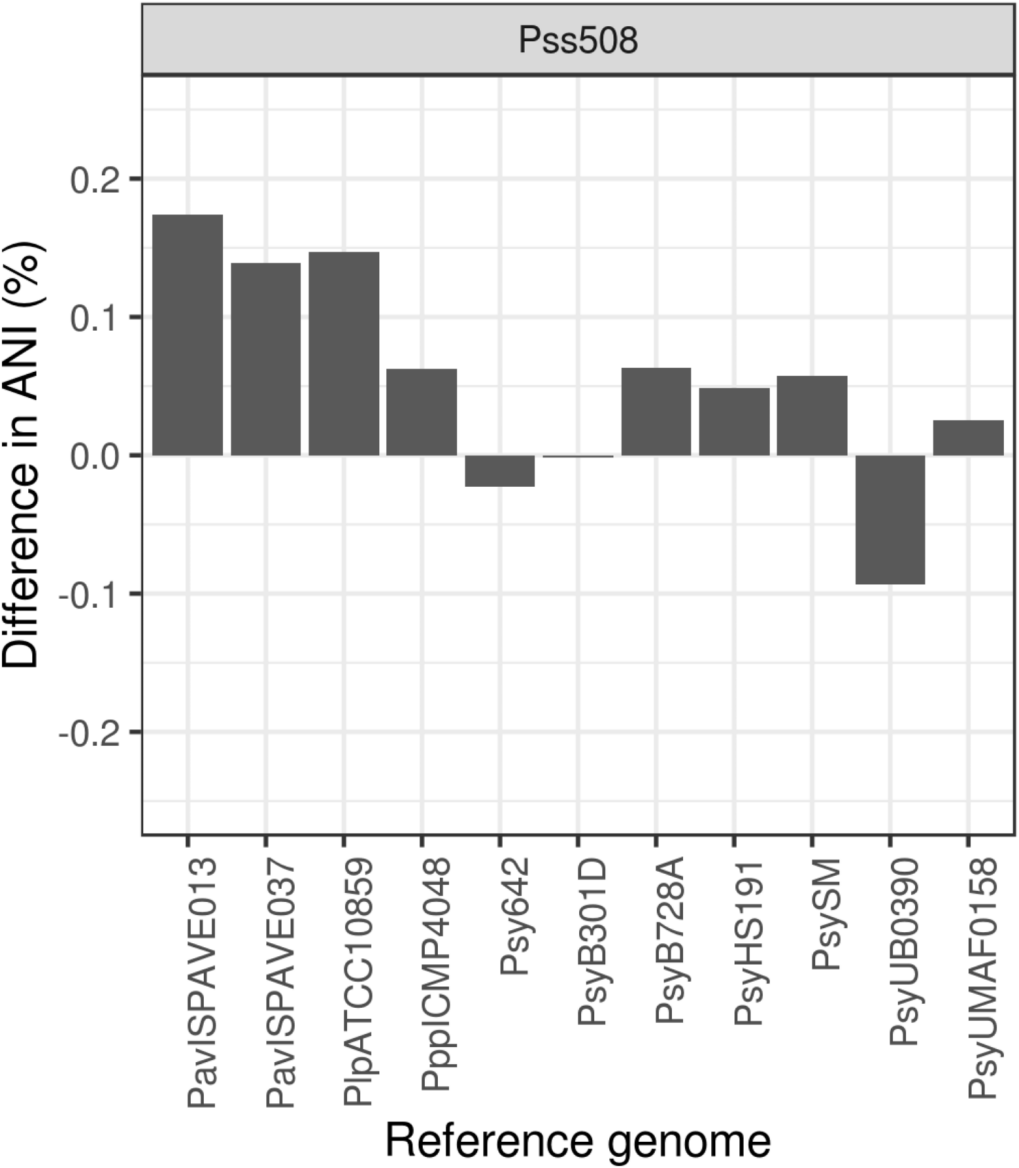
Post Kastor correction Average Nucleotide Identity (ANI) percent differences. ANI between Pss508 and reference genomes were compared before and after error correction

Many of the detected insertion errors follow either adenine or thymine but are not associated with any particular homopolymer (Fig. 2). Although insertion errors are more frequently associated with homopolymers of length two and five, this trend differs for PsyTLP2 (Supplementary Tables S1-2 and Figs. 1, 2 and 3) indicating some variation between sequencing runs. Unexpectedly, two of the top four 4-mers for insertions are not homopolymer repeats. Both CCCA and TCCA, as well as ACCA (#9), are two or more cytosine followed by a single adenine and a second erroneous adenine. The combination of these 4-mers likely contributes the majority of errors associated with shorter homopolymers. This suggests that there is potential difficulty discerning the number of adenines following a nucleotide run of cytosines. Collectively, the high abundance of homopolymer containing k-mers is indicative of non-random errors in minION long read assemblies associated largely with homopolymer regions [10, 19].

### Non-corrected pseudogenes

Since Kastor candidate error correction is dependent on reference information, a lack of reference genome coverage can limit the error detection leading to possible false negatives and consequently non-corrected genes. For *Pseudomonas syringae* related strains, Dillon and colleagues (2019) report that the core genome (2410 genes) of greater than 400 Psy (*sensu lato*) genomes represent slightly less than 50% of a typical Psy genome assembly [2]. Reliable reference coverage of the remaining genome is contingent on the user’s choice of reference genomes due to an expansive accessory genome reflective of the species high diversity [42]. Using the primary equidistant reference set (set one), approximately 10% of the Pss508 assembly had insufficient reference genome coverage (< 5 genomes) to confidently search for error candidates.

Within the Pss508 assembly, 953 genes annotated as incomplete or frameshifted were corrected, and 296 frameshifted or incomplete genes were retained in the post-Kastor assembly (Table 3). For comparison, 233 frameshifted or incomplete genes are present in the post-correction Psy642 Illumina assembly, with a larger proportion of incomplete genes. Within both genomes a proportion of the frameshifted or incomplete genes are likely true pseudogenes, however the greater proportion of frameshifted genes in the Pss508 assembly suggests that many of the non-corrected frameshifts may represent retained indel errors. We investigated the role of reference genome coverage on these non-corrected pseudogenes. Excluding the four newly annotated pseudogenes and four previously complete genes, 283 of the remaining 288 pseudogenes in Pss508 did not contain detected errors (Table 3). We determined 148 (52.3%) of these pseudogenes did not have sufficient coverage for at least 50% of the gene. These genes were also found in specifically low coverage regions. Notably, the median gene length for many of these pseudogenes is well below an average bacterial gene [43]. Upon manual inspection, some of the corresponding reference genes have since been depreciated in NCBI’s database suggesting that a subset of these short genes may be spurious annotations [44].

### Optional maximizing of error correction

To assess the impact of increasing reference genome coverage on error correction, we also tested Kastor to Pss508 with an additional two reference genome sets that have an expanded number and diversity of Psy strains (Supplementary File S4). We wished to test whether increasing the pool of available reference genomes could improve error detection by maximizing coverage of the accessory genome by introducing more divergent sequences (Pseudomonas sets two and three) [45]. Since *Pseudomonas syringae* encompasses an entire species complex with diverse taxonomic phylogroups, we grouped the Pseudomonas reference genomes based on degree of similiarity. Our set one data (main reference set, 11 genomes) includes a curated number of equidistant genomes at the species-level but is limited in the number of genomes used (Table S5). Set one should encompass the core and a representative portion of the accessory genomes. Set two is non-curated and comprised of 170 genomes within the Group 2 Psy phylogroup, the closest group to main test dataset (Pss508, PsyTLP2, and Psy642). These genomes should contain a representative core and accessory that should be found in our test data set. Set three is a non-curated species-level reference genome set, which includes all 441 available strains from all Psy phylogroups [42]. This set should contain the largest accessory genome with most known genes to *Pseudomonas syringae* allowing maximizing possible coverage of the target draft assemblies.

For both sets two and three, the error threshold was increased to 0.995 and 0.999 to ensure a consensus across the higher number of genomes. These genome sets marginally improved the target assembly coverage with ~ 97% and ~ 98% of Pss508 determined to have sufficient reference genome coverage respectively. Improved coverage was found in the accessory genome especially among highly mobile genes. Additional coverage was minimal in set three compared to set two, indicating that the majority of accessory genes are typically found in closely rather than distantly related strains. Despite the higher coverage in both cases, when these corrected assemblies were assessed using BUSCO, they were found to have an additional 11–18 universal orthologs shown as fragmented indicating slightly poorer correction when compared to using the equidistant reference dataset (set 1) (Table S7). Despite sufficient coverage of core genes, there was less sequence consensus due to increased nucleotide variability between more distant genomes. This variation leads to lower error detection in previously covered regions using reference genome set one.

To offset the tradeoff between higher coverage and detection of very conserved assembly errors, we implemented an optional hybrid approach where Kastor can take advantage of multiple genomic sets with a different diversity of sequences. A primary reference set can be supplemented with reference information from the secondary genome set only in regions of low coverage. This strategy allows for coverage in the distant accessory genome while maintaining the higher candidate detection rate of the primary set. We tested the effect of three different combinations of multiple reference genomes sets. In all cases, the primary set was the equidistant 11 genome set (set one). This set was either supplemented with no additional reference genomes, the group 2 psy set (set two), or the psy 441 set (set three). When using a combination of reference sets, an additional 253 and 177 errors were detected in the Pss508 long-read assembly (Table S8), with the majority of these errors appear within genes in previously low coverage regions. Correction of these errors was beneficial in defragmenting an additional ~ 50–60 pseudogenes. Overall, a single, well-curated reference genome set is sufficient for Kastor to correct the majority of assembly errors, but error correction can be maximized with an additional curated genome set with a slightly higher diversity of genomes.

### Reference genome bias on post-correction assemblies

A concern about using reference information is biasing the correction of the final assembly towards one or a small group of reference genomes. Even after implementing a strain-specific read-based adjustment, greater than half of detected candidate indel errors lacked any read support for target strain specific corrections. Here, Kastor relies solely on reference alignment differences to determine the error site and suggested correction. Since the error site for a region is determined by scoring all possible sites, it is possible for an error site originating from a single or group reference genome to be consistently scored higher. Without any read-based adjustments, in theory these corrections would move the target assembly closer towards those reference genomes. Successful de-fragmentation, with minimal bias, should improve assembly similarity to the closely related *P. syringae* genomes in the reference set, especially for conserved genes. To determine if a potential bias towards specific genomes existed, we compared each pre- and post-correction assemblies to the reference *P. syringae* genomes in the equidistant genome dataset.

Sequence similarity was assessed by comparing the average nucleotide identity (ANI) between each assembly and each reference genome using orthoANI [46]. As expected, comparing the post-corrected Pss508 to Psy642 and PsyUB0390 (the most closest related strains) had ~ 98% ANI which is consistent with strains belonging to phylogroup 2c [2]. All other *P. syringae* strains had an ANI of ~ 94% to the two assemblies, which is similar to distance from Psy642 to the other strains (Table S5). Comparing ANI differences between pre- and post-correction assemblies, the shift in ANI was less than 0.3%, as expected from the low rate of corrections (Fig. 3). In fact, ANI increased for almost all strains when compared to Pss508. Unexpectedly, one exception is that the Pss508 assembly decreased slightly in similarity to both group 2c strains. Notably, there is an increase in similarity to *avellanae* pathovar strains for Pss508. A correction bias towards *avellanae* pathovars is less likely when the second highest ANI increase was with a *P. syringae* pv. *Lapsa* strain which is more distant to the *avellanae* pathovars than other strains [2]. Overall, Kastor corrections do not appear biased towards any particular strain or group and have minimal impact on nucleotide similarity to reference strains.

## Discussion

### Number of detectable errors is inversely proportional to draft assembly quality

Kastor was designed and intended to detect assembly errors found in draft assemblies using reference genomes in the public space as a point of comparison. We hypothesized that functionally important putative assembly errors such as gene-fragmenting errors would be very rare in all references. Consequently, the number of errors is reflective of the overall sequence quality within draft assemblies and is supported by our test data set. With Kastor, the greatest number of errors were found in long read assemblies generated using older sequencing chemistry, Pss508 (34.23 indels/100 kb), PsyTLP2 (5.205 indels/100 kb), and PfW2 (187.36 indels/100 kb), followed by the hybrid assembly, CkMINF_9D (1.7 indels/100 kb). Unsurprisingly, the fewest were found in Q20 + long read assembly Mtb (0.13 indels/100 kb) supporting major quality improvements reported in later iterations of nanopore sequencing [16, 17]. Therefore, using reference genomes, Kastor’s detected errors can be used as an estimate of assembly quality, and also act as an assembly polisher, improving assembly quality to a level comparable to short read polishing without additional sequencing.

### Considerations and limitations in error detection accuracy

Ideally, a draft assembly with high sequence quality would have minimal corrections reflecting a low rate of introduced errors. We assessed the performance of Kastor in error correction by comparing the errors detected between two closely related *P. syringae* draft assemblies, the Pss508 long read assembly, and the Psy642 Illumina short read draft assembly. In both cases the same reference set was used in error detection allowing for direct comparison. As the current standard for low error sequencing, we expect the Illumina sequence to have minimal nucleotide errors and can serve as a baseline for background error correction [4]. Kastor detected many more errors in Pss508, with up to 15x more errors than the Illumina equivalent Psy642 (Table 1). However, Kastor also found 195 potential assembly errors in Psy642 suggesting some errors may be present despite a higher sequence quality. Alternately, if we assume the Illumina sequence assembly represents a reasonable ground truth, we can estimate an upper false positive limit of one erroneous correction per ~ 29,800 basepairs. This rate can then be defined as the upper limit since Illumina sequences may still contain errors that require correction, e.g. some errors may be true positives [47].

Unlike in the Pss508 assembly, the majority of indel errors (91%) were found in intergenic regions of Psy642 (Table 1). Presumably these errors would include false positives due to lower sequence conservation in intergenic regions. This higher variability can lead to poor alignment and spurious error detection. A false positive rate is difficult to quantify even with detected intergenic assembly errors in an Illumina assembly since 18–32% of intergenic regions greater than 60 nt were found to be conserved in a variety of bacteria [48]. These conserved regions can contain sRNAs, ncRNA, and regulatory elements under evolutionary pressure [49, 50]. This sequence conservation suggests that poor alignment is unlikely to introduce spurious error detection and that some of these intergenic errors are true positives. In contrast, variable length homopolymeric repeats in small RNAs have also been implicated in regulatory function [51], and it is challenging to separate true and false positives in these regions.

Although Kastor is effective in detecting putative assembly errors, it is reliant on accurate and precise mapping of the reference genomes, influencing both false positive and false negative rates in error detection. This reliance is affected by three main factors: reference genome selection, reference quality, and mapping quality. Therefore, a potential limitation to Kastor is the availability of high-quality reference genomes for selection. Inclusion of highly divergent genomes can increase genomic coverage of accessory genes that would be missed with a limited reference set (Table S8). Conversely, high diversity reference sets can introduce too many variations and can mask clade specific mutations even in universal orthologs hindering detection sensitivity [42]. In other cases, in tests using a higher taxonomic level reference dataset (i.e., genus) more errors were detected than those with lower taxonomic level equivalents leading detection of possible variants in the target species, particularly in intergenic regions. These low amounts of additional errors do not appear to negatively impact the assembly.

The chosen genome set used should still be sufficiently diverse to not bias correction towards any particular reference. In ideal cases, we recommend a reference genome set of around 10 closely related but approximately equidistant reference genomes that belong to the same monophyletic groups as the target organism. If available, including at least one sister strain (~ 98% identity) is also recommended to reduce potential false detection of single variants. The genome collections used here were sufficient to detect the majority of assembly errors (Table 1). An optional supplementary reference set with greater number of more divergent sequences may be provided to increase coverage and maximize error detection but is not required as the gain in accuracy is not pronounced (Table S8). When provided with a secondary set, there was slight improvement to the Pss508 assembly with an additional 68 complete genes (~ 7%).

Unfortunately, stringent reference curation is not feasible for many organisms. Under non-ideal conditions, a reduced number of reference genomes can still be used, as suggested by the tested *Plasmodium falciparum* draft assembly (Table 2). With only a singular complete reference genome, supplementing the reference with lower quality (i.e., draft, scaffolds) assemblies can still provide sufficient error detection. One caveat is that missing sequences in the lower quality reference assemblies can lead to lower coverage of the assembly and consequently missed errors. With PfW2, only errors associated with orthologs present in scaffolded assemblies were detectable. Upon correction, PfW2’s assembly completeness only reached scaffold level when compared to the complete reference. In other cases, reduced continuity in draft references can introduce spurious regions of higher coverage from overlapping contigs or regions of low coverage [4, 5].

Although Kastor was intended to be organism-agnostic, one possible future consideration is the implementation of a pangenomic approach to error determination. Use of a pangenome graph can be used in identification of representative allele variants per error candidate [52]. For each search window, reference bias can then be reduced from more distantly aligned, thus non-representative, reference assemblies while still maintaining reference coverage in more divergent accessory genes. In other words, this approach could reduce both false positives, via consideration of more representative sequences, and false negatives, via expanding coverage by considering more reference genomes.

When references are limited, our generalized approach is still effective for error detection. For instance, if user intent is error detection only and not correction, two to three closely related references should be sufficient as a minimum number. Otherwise, currently a well-rounded and high-quality genome set can be most effective in the assessment of errors present in long read assemblies, and thus careful selection of reference genomes is recommended.

## Conclusion

Kastor was capable of effectively detecting errors in all tested small genome long-read draft assemblies using reference genome information. Upon correction of these errors, assemblies were sufficiently accurate and complete for effective gene annotation [28]. This form of guided error correction can be used as an alternative method of final polishing without the need for additional short read sequencing [3, 11]. Additionally, our software can be used to estimate the prevalence of small indel errors in long-read assemblies [19]. Such estimates may provide researchers with a useful benchmark to compare assembly quality across different assembly pipelines.

## Methods

### Design overview

**Fig. 4:**
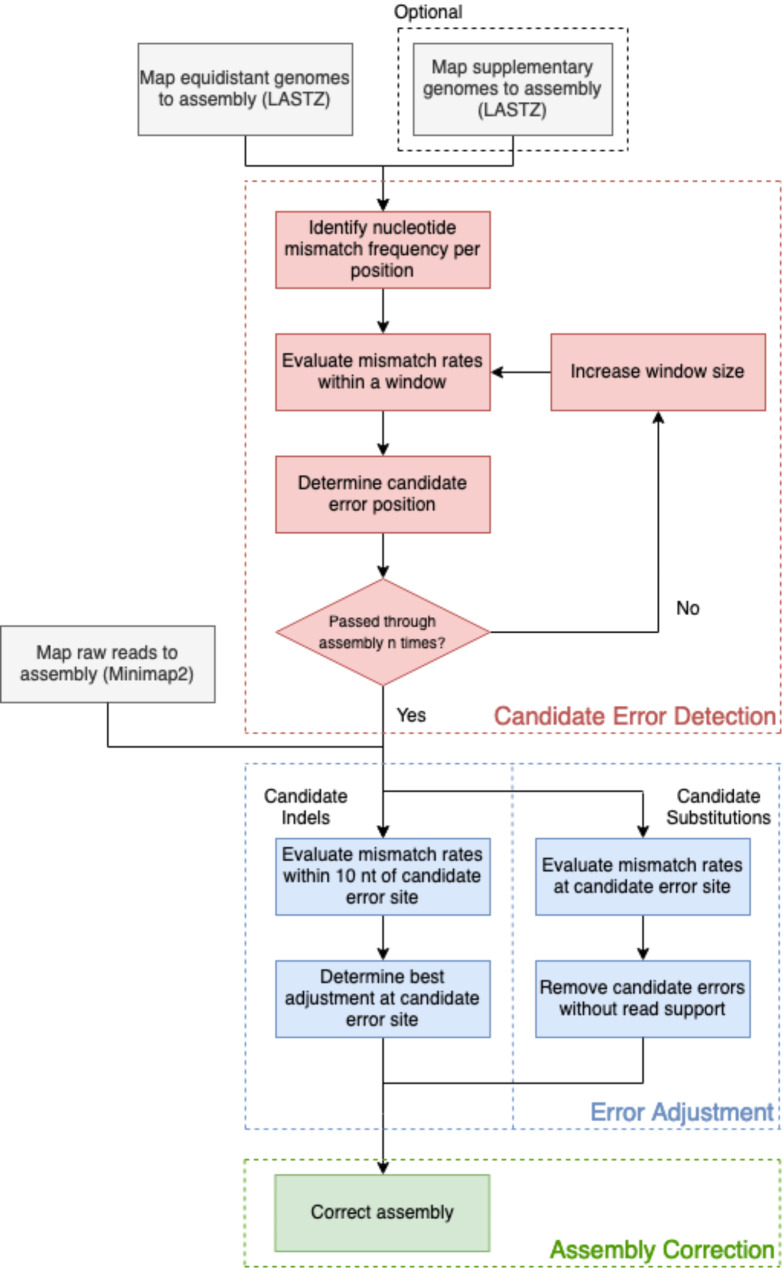
Kastor error assessment and correction workflow. Each module in Kastor is highlighted as a separate colour: Red – Error detection, Blue – Error adjustment, Green–Assembly Correction. External steps in the workflow are indicated in grey

Kastor is a reference-based correction software written in Python and is separated into three main modules: reference-based detection of candidate errors, error adjustment and confirmation using raw read information, and assembly correction (Fig. 4). The first module is designed to compare the draft assembly with locally aligned reference genomes to identify candidate sites for assembly errors. Candidate sites are then compared with aligned raw read data to adjust or confirm previously detected error candidates with sufficient read support. Read support is used to minimize reference bias and retain strain variability, within range of the candidate errors. For indel errors, nearby read-supported error equivalents are favored over the initial candidate and the detected error site is adjusted accordingly. The third module uses both the error information and suggested corrections to correct the corresponding draft assembly.

### Bacterial strains, reference genome selection, and pre-processing

Two plant-associated *Pseudomonas syringae* strains [28], *Pseudomonas syringae* 508 (Pss508) and *Pseudomonas syringae* TLP2 (PsyTLP2), were sequenced using ONT’s MinION sequencing technology [53]. These strains were chosen as our main test data for Kastor assessment as two complete long read only assembly lacking supporting supplemental short read sequencing. Both genomes were assembled to their pre-correction assembly using an overlap-layout-consensus pipeline comprised of Minimap2 (v2.10-r761), Miniasm (v0.2-r159-dirty) and Racon (v1.4.3), and then polished with Nanopolish (version 1.6) and Medaka (v0.2.0) [10, 24, 25, 54, 55]. To confirm assembly improvements by Kastor error correction with alternative and recently developed software, these sequencing runs were re-basecalled using Dorado (v0.8.0) [40], assembled using Flye (version 2.9.5) [26], and polished using Medaka (v2.0.1) [25] to test assembly completeness. The previously published genome for the closely related strain *Pseudomonas syringae* 642 (Psy642) was also used as an Illumina assembly for comparison [53] and is expected to have relatively few indel errors.

Three different sets of *P. syringae* (Psy) genomes with varying diversity and number of genomes were retrieved from the NCBI genome database and used for reference-based assembly error correction (Supplementary File S4). Each set targets a different taxonomic level. Set one is a high-quality collection of 11 closely related Psy strains with an assembly quality classified as scaffold or higher, with the exception of Psy642. The strains for this primary set were selected to have approximately equal phylogenetic distances to the target strain and each other based on both gapped (94–98%) and symmetrical (78–82%) identity as provided by NCBI (Table S5). To minimize bias towards any particular taxonomic groups, groups with higher shared gapped identity (~ 98%) were limited to three strains. Genome sets two and three include a greater variety of non-curated draft genomes. Set two includes 170 Psy strains (*sensu lato*) that belong to phylogroup 2, the closest related monophyletic group [42]; set three includes a selection 441 Psy genomes predominately classified as genomespecies 1 in NCBI and select strains from most of the 13 broad monophyletic groups, i.e., most available psy strains taxonomically close to our target strains.

Each reference genome set and the target strain raw reads were aligned to the target assembly using LASTZ (v1.04.03) [56] with a minimum identity of 80%, no chaining and no interpolation. Poor alignments and short alignments (< 1000 nt) were filtered to minimize spurious reference mapping. The full alignment was then converted to an mpileup file (Samtools v1.2) using the–aa flag to ensure that all zero coverage positions are reported within the file to maintain consistency between the alignment files.

For assembly polishing comparisons, bacteria assemblies for *Citrobacter koseri* and *Mycobacterium tuberculosis* were included in our test dataset. *C. koseri* is a representative hybrid draft assembly, assembled using Trycycler and polished with Medaka, and polypolish corrected version were available from its past study [33]. *M. tuberculosis* is a representative long read assembly generated using improved nanopore chemistry (Q20+) and was assembled with the standardized assembly pipeline with Flye (v2.9.5) and Medaka (v2.0.1) [57]. These assemblies were corrected using reference genomic information at either the genus (*n* = 8) or species (*n* = 11 to 15) level. Average nucleotide identity between reference genomes were used to reduce groups of highly similar reference genomes similar to psy reference selection. Reference genomes were mapped as mentioned above. Polypolish-corrected *C. koseri* was available from [33], and FMLRC2 (v0.1.8) was used on *C. koseri* genes using short reads provided by the respective studies [33, 34, 35]. The former was provided by the study itself and was not re-run. Due to high similarity in results, only data from polypolish corrected assemblies are shown.

To demonstrate that Kastor error detection is organism-agnostic, we also included a *Plasmodium falciparum* draft assembly in our test data as a representative small eukaryotic genome. At the species level, only one complete reference genome was available. To supplement the reference set, we selected an additional five reference genomes, complete at the chromosome assembly level for a total reference set of six genomes. Unlike the bacteria test dataset, we increased LASTZ (v1.04.03) sensitivity to mapping by reducing the hsp threshold and allowed for sensitive interpolation (K = 2400, L = 3000, inner = 2000).

### Software implementation

#### Module one: candidate error detection

For each contig, the software cycles through each position and assesses whether there is sufficient reference genome coverage (≥ 5 genomes) to infer a candidate error site. At sites with sufficient depth, Kastor calculates the frequencies of substitutions and indels in reference genomes as compared to the target genome, which are then used to calculate an alignment difference probability (ADP) for each error type. ADP are calculated as the proportion of reference genomes that have a difference with the draft assembly at the current site. The ADP for substitution errors is based on the frequency of nucleotide mismatches. For insertion errors, Kastor calculates the proportion of reference genomes missing sequence(s) at the position (i.e., a “deletion” in the reference genome). For deletion errors, the inverse is considered. The software uses this ADP for the initial identification of potential sites for error correction. When the probability of a putative error equals or exceeds the error threshold set by the user (default = 0.99; i.e., 100% of reference genomes must exhibit the difference), the position is identified as a candidate error site. For example, if a G at position X in the draft assembly aligns with a highly conserved A in all the reference genomes, the ADP is calculated as the proportion of reference nucleotides at position X that are not G. In the case where the ADP exceeds the error threshold, the substitution G to A at position X is classified as a candidate error.

The ADP is calculated and evaluated independently for substitutions, deletions and insertions. As longer indels are likely more reflective of a genome-specific variant of the sequence rather than a candidate assembly error, candidate insertion and deletion errors greater than two nucleotides are ignored. To avoid collisions with longer indels, the ADP is calculated only at the starting position of the alignment indel difference. For example, if some reference genomes are missing nucleotides AG at positions Y, Y + 1 in the draft assembly, an insertion ADP of < 1.0 is calculated for position Y only and considered as a single putative insertion error two nucleotides long.

The software then scans through saved alignment information with a minimum of two and a default of four elective iterations. These iterations are intended to disambiguate highly variable regions containing multiple candidate errors in close proximity. The software attempts to reduce the complexity of the region by first flagging simpler cases in earlier passes then disambiguating additional cases in subsequent iterations (Figure S5). This is achieved by varying the window size in each iteration. The first iteration has a window size of one and in the second iteration the window size varies with homopolymer size. The third pass (if used) has a starting window size of 11, where five nucleotides flank each side of the candidate site. This window increases by five nucleotides each side for subsequent iterations.

During each pass, Kastor evaluates the ADP for all error types at each position and retains the greatest ADP. In the infrequent case of a combined substitution error and deletion error, this would be parsed as two adjacent errors in the first and second mandatory pass. If the ADP exceeds a threshold of 0.25 (i.e., 25% of aligned reference genomes contained alignment differences), the position is considered as a candidate error location. The software then calculates a regional ADP for the set window size. During this stage, Kastor keeps track of which sequence contributed to the regional ADP rate to ensure that multiple differences on a single aligned sequence cannot contribute to the regional rate.

To maximize error finding, Kastor also employs two additional search strategies that are limited to elective passes at every second pass. The first strategy shifts the search window left and right while maintaining window size to facilitate detection in situations where the candidate site is not central to an erroneous region. The second strategy is the consideration of different error lengths for the same error type. The software defaults to considering different error lengths (i.e., indels of one and two) as independent errors to simplify early detection. With this search strategy, a putative two-nucleotide deletion can be treated as a single deletion. This is intended for cases where there is a disagreement in error length rather than a presence of a candidate error.

If the ADP exceeds the error threshold, the software then evaluates the region to identify potential erroneous nucleotide(s). Kastor scores each candidate location containing a reference alignment mismatch as a putative true state, where the score = (mismatch frequency x homopolymer length). If multiple locations belong to the same homopolymer and are of the same correction type, the sites are merged before score calculation. Since Kastor focuses on errors found in long read assemblies where many indel errors are associated with homopolymers, the score is weighted slightly towards mismatches involving longer homopolymers [15]. However, if the alignment difference suggests a correction that would split a homopolymer, the score is penalized by half. The candidate position with the highest score is considered the candidate error site for correction.

#### Module two: error adjustments

The second module aims to use target genome read data to support candidate error locations and minimize bias towards reference genomes (Fig. 4). An alignment of the sequence data to the draft assembly is required. For every indel error, Kastor sets a 21-nucleotide window with the candidate error position at the center. Similar to the first module, a read ADP of the respective error type is calculated for every position in the search window. Adjacent repeat nucleotides are grouped into a single representative candidate site to prevent distributing potential errors over multiple equivalent positions within a homopolymer. An exception to this is an error that introduces a break within a homopolymer, which is considered as a unique and independent position. Any position with a read ADP exceeding the user-defined adjustment error threshold is considered a read-supported candidate error site(s) for adjustment.

Kastor assesses all read-based candidate sites for support of the initial reference-based candidate error prior to any evaluation of alternative candidate error sites. We reason that with sufficient support for the initial error candidate no adjustment is needed, and alternate read-based candidates are not evaluated. In cases where the initial candidate has no read support, Kastor considers the previously identified read-supported candidate error sites as possible alternate candidate locations for the detected error. The alternative with the greatest read ADP is selected to replace the initial reference-detected error candidate site. In the event that no candidates of the same length are found, multiple smaller error candidates may be considered for adjustment. More specifically, an indel candidate of 2 nucleotides may be adjusted to become two single indels if there is read data support. However, a lack of any or insufficient amounts of shorter supported candidates will result in no adjustments.

In contrast, substitutions are evaluated differently. From module one, unlike indels, all possible substitutions are considered and evaluated for read support. For example, a candidate site with both adenine and guanine mismatches in the reference alignments will consider both nucleotide as a possible correct state. For the same position, Kastor calculates a read ADP for all possible nucleotides and finalizes the candidate substitution correction with greatest ADP that exceeds the adjustment threshold as the likely true state. Unlike indel errors, this adjustment module searches for read support only for substitutions. A lack of support will result in the removal of the substitution as a detected error. Once all candidate error adjustments are made, the finalized list of errors are passed to the third module for assembly correction, unless specified as an output.

#### Module three: assembly correction

The finalized list of errors is then used to correct the assembly and output a fasta file. If desired, users can manually inspect the error correction list as a tab-delimited output file. This error list may be used as input to reproduce the assembly correction without re-running the program.

### Correction and analysis

Completeness of the corrected assemblies was analyzed using BUSCO (v7.5.1), a software that benchmarks the status and copy number of single copy universal orthologs [38]. Gene fragmentation of the pre and post-correction assembly was analyzed using NCBI’s PGAP (build 7555) [41]. The RefSeq protein set used in the gene annotation was used to assess whether improvements are primarily in gene de-fragmentation or whether percent identity to reference genomes is also altered. NCBI’s tblastn was used for all comparison of RefSeq annotated proteins to pre- and post-corrected assemblies. The analysis of pre- and post-corrected assembly relatedness to reference genomes was assessed using orthoANI [46]. All tables and plots were generated using R version 3.4.2 and Rstudio version 1.1.383 using packages dplyr (v0.7.8), tidyr (v0.7.2), ggplot2 (v2.2.1) and ggpubr (v0.1.8).

## Electronic supplementary material

Below is the link to the electronic supplementary material.

Supplementary Material 1

Supplementary Material 2

## Data Availability

Sequence data for Pseudomonas genomes used in this work is available at NCBI under BioProjects PRJNA449945, PRJNA449517, and PRJNA40347, and raw reads for Psy assemblies are available at SRA archives SRR11658055 and SRR11658508. Kastor is distributed freely. Project name: Kastor. Project home page: https://github.com/Waterloo-McConkey-Lab/Kastor. Operating systems: Unix-based 64-bit OS. Programming Language: Python 3. Other requirements: None. License: GNU GPL. Any restrictions to use by non-academics: Non-academics may freely use this software.
