# Supplementary material for: Kastor: a reference-based comparative approach for assessment and correction of gene-fragmenting errors in long-read assemblies of small genomes
Source: BMC Genomics. 2025 Apr 18;26:388. doi: 10.1186/s12864-025-11569-y (PMC12007338; doi:10.1186/s12864-025-11569-y)
Supplement: Supplementary file 1 — Supplementary Material 1 [file 12864_2025_11569_MOESM1_ESM.docx]

**Supplementary Figures**


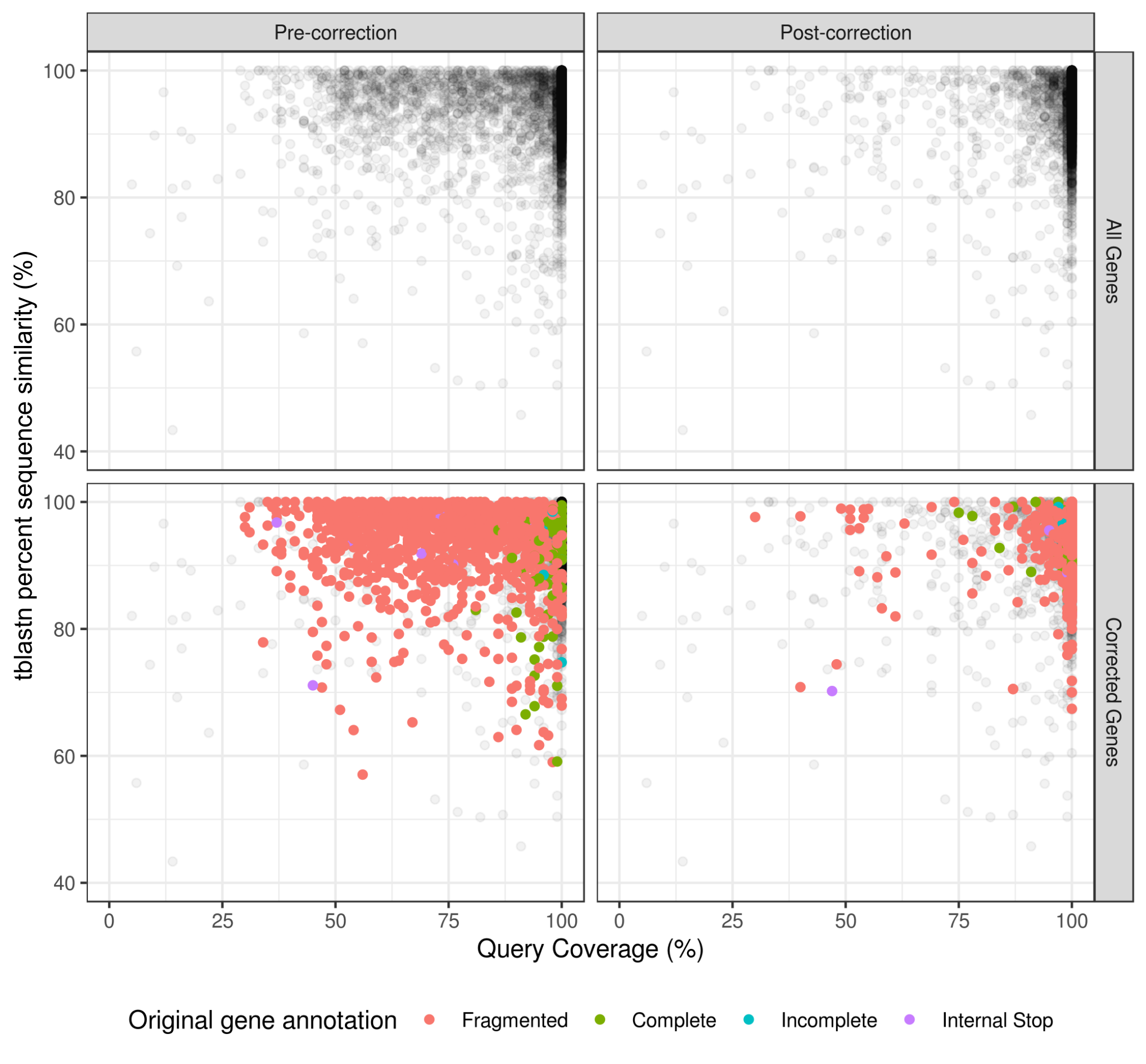


Figure S1. Reduction of fragmentation in identified genes before and after Kastor correction. (Top) Overall profile of gene estimated from RefSeq proteins mapped back to PsyTLP2. (Bottom) Changes in genes found before and after correction. Genes containing corrections are highlighted by the original annotation. RefSeq proteins used in PGAP’s protein homology annotation of pre-corrected assemblies were mapped back to each assembly using tblastn. Corrected genes are colored according to the original annotations while non-corrected genes are represented as grey. In detail, fragmented indicate genes where two or more frameshifted gene fragments were found; complete indicates complete genes; incomplete indicate truncated genes at the 5’ or 3’ end; and internal stop indicates genes containing a stop codon truncating the full gene. Gene fragmentation or incompleteness is inferred from lower query coverage when tblastn detects only a fragment of expected gene. Longer and more similar genes were found after correction.


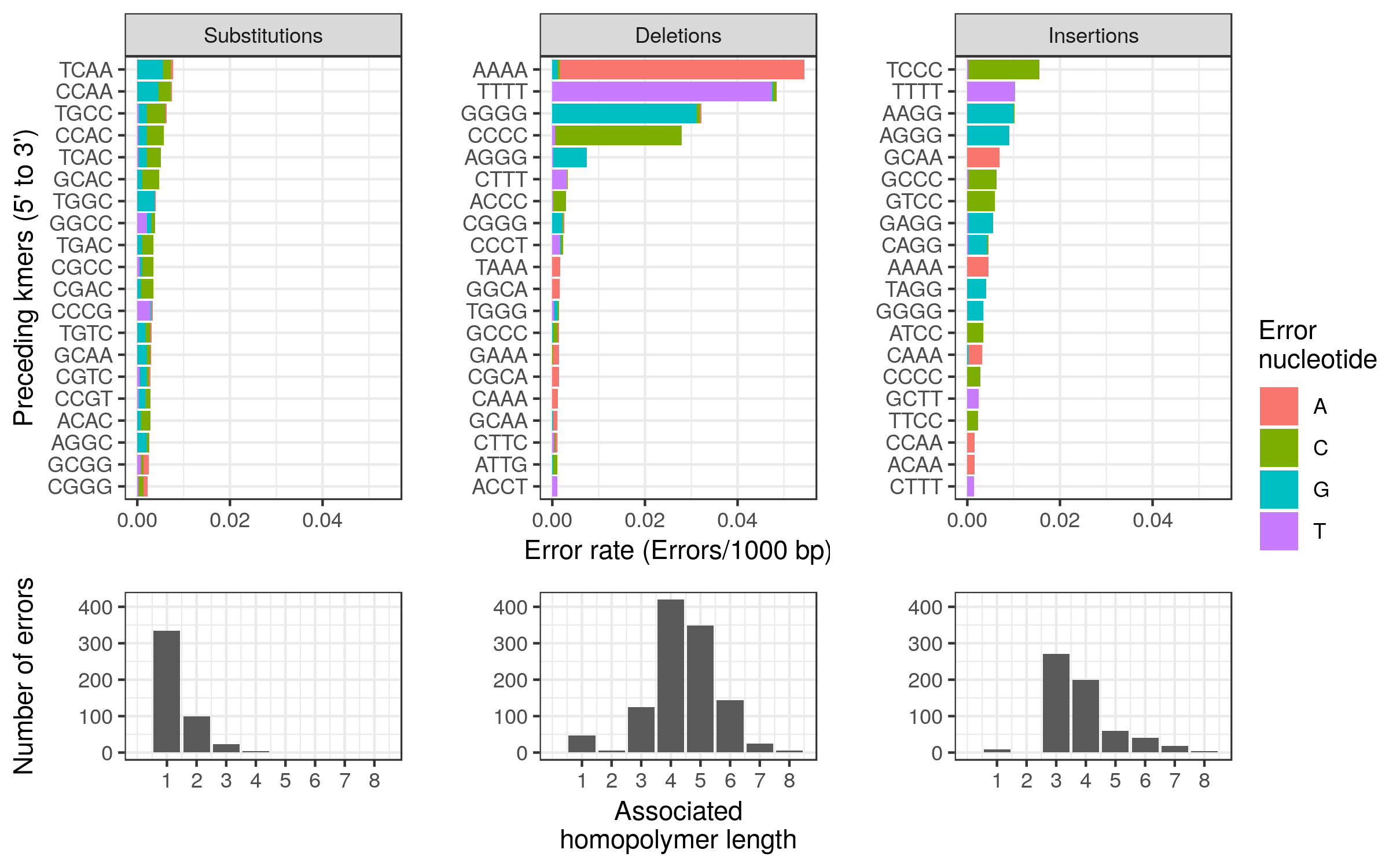


Figure S2. Breakdown of the nucleotide sequence preceding detected errors in the PsyTLP2 assembly. Top plots: Top 20 k-mers (k = 4) upstream of detected errors separated by error type. Each bar is colored with the associated erroneous nucleotide. Bottom plots: the distribution of the length of upstream homopolymers for the respective error type.


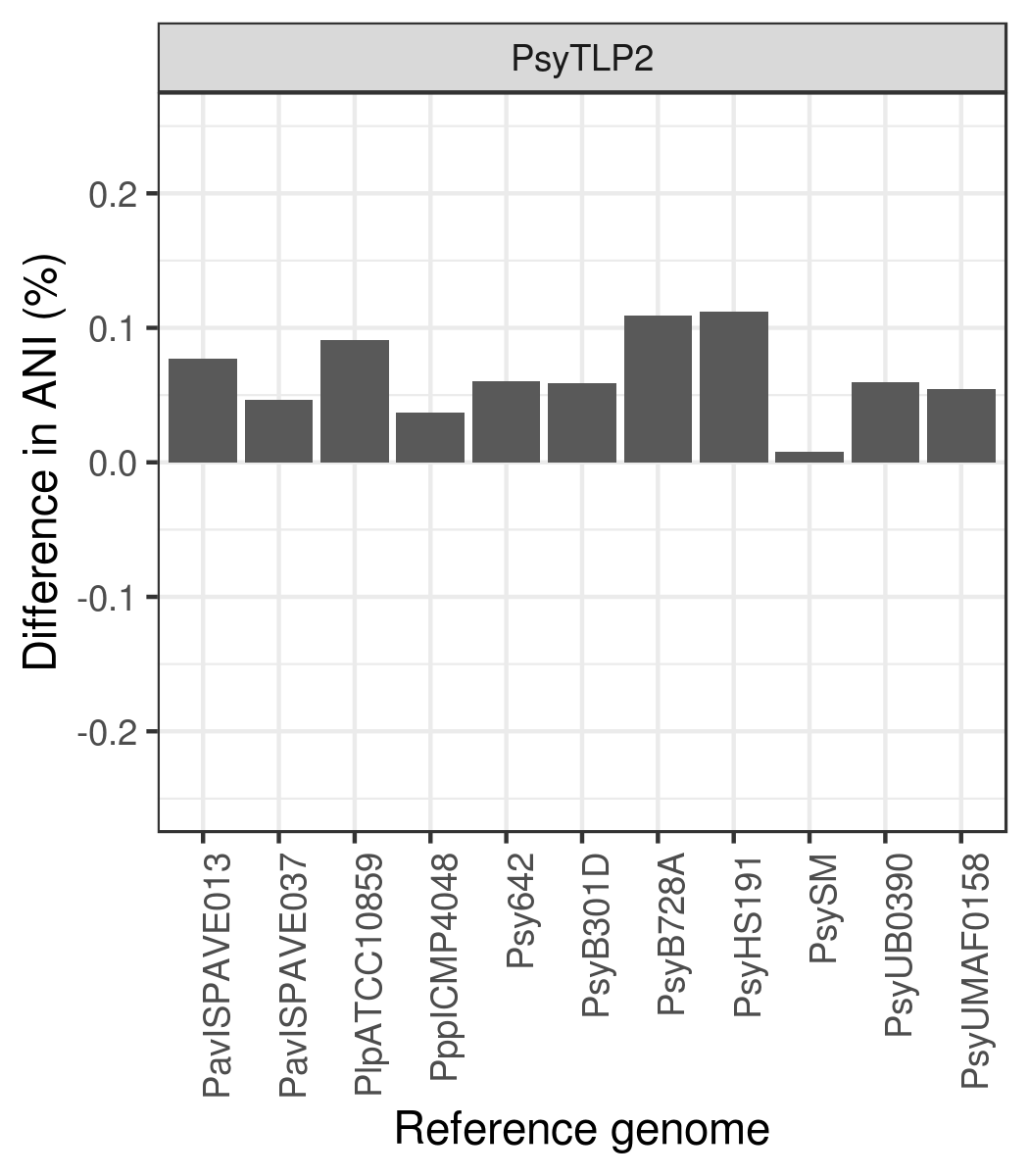


Figure S3. Post Kastor correction Average Nucleotide Identity percent differences. ANI between PsyTLP2 and reference genomes was compared before and after error correction.


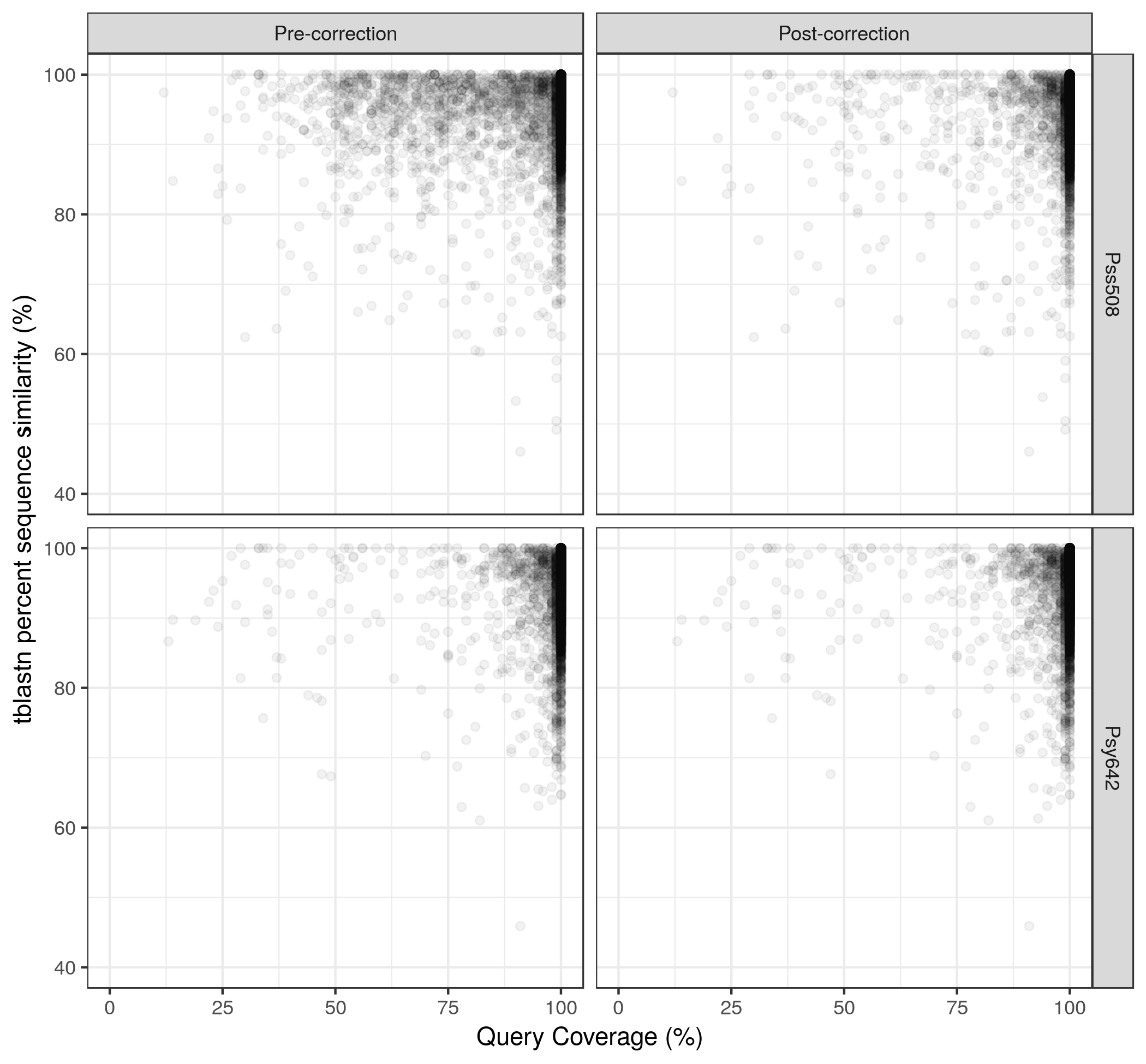


Figure S4. Expected gene profile in post-corrected assemblies. The proteins used in PGAP’s protein homology annotation of the respective assemblies were mapped back to each assembly using tblastn to determine the equivalent genes. High query coverage and similarity to RefSeq proteins are indicative of highly similar genes encoding known proteins.


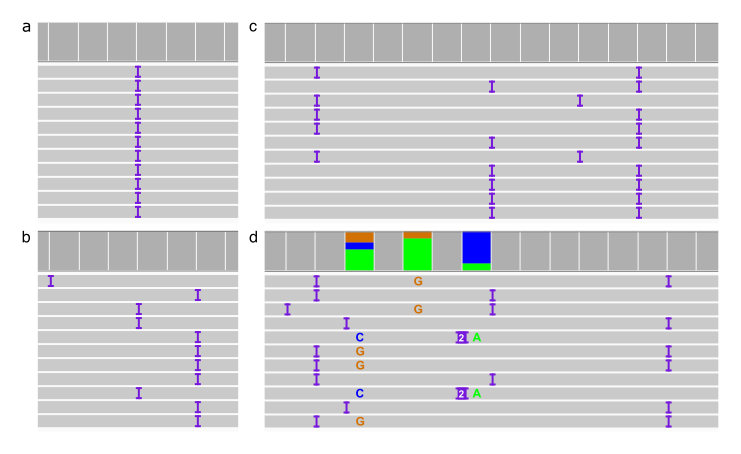


Figure S5. IGV visualized examples of draft assembly regions (Pss508) containing deletion error candidates. The topmost track represent single positions (grey/coloured blocks) in the draft assembly. Each subsequent grey track represent an aligned reference genome. Visible nucleotides are mismatches while purple indicators represent unaligned additional nucleotides found in the reference genome. a) Simple case. Single position with an adjacent deletion error candidate. b) Single deletion error with variable candidate sites. c) A region with two deletion error candidates in close proximity. Smaller windows in earlier passes disambiguate the region as two simpler error candidates side by side. d) Ambiguous region with either two single nucleotide or one double nucleotide deletion error candidate.

**Supplementary Tables**

Table S1**.** Summary of the small genome test assemblies used in this study (<22Mbps). One round of long read polishing using Medaka was run on all long read and hybrid assemblies.

| **Organism** | **Flowcell** | **Basecaller** | **Assembly type^1^** | **Source** |
| --- | --- | --- | --- | --- |
| ***Pseudomonas syringae* 508**  ***Pseudomonas syringae* 508 (DFM)^1^** | R9.4.1 | Albacore | Long read only | This study |
|  | R9.4.1 | Dorado | Long read only | This study |
| ***Pseudomonas syringae* TLP2** | R9.4.1 | Albacore | Long read only | This study |
| ***Pseudomonas syringae* TLP2 (DFM)^1^** | R9.4.1 | Dorado | Long read only | This study |
| ***Pseudomonas syringae* 642** | - | Illumina | Short read only | [53] |
| ***Citrobacter koseri* MINF_9D** | R9.4.1 | Guppy | Hybrid | [33 - 34] |
| ***Mycobacterium tuberculosis*** | R10+ | Dorado | Long read only | [57] |
| ***Plasmodium falciparum* W2** | R9.4.1 | MinKNOW | Long read only | [39] |

1 – For testing of more recent assembly pipeline, dataset was re-basecalled using Dorado and assembled using Flye and Medaka (see Methods)

Table S2**.** BUSCO assessment of assembly completeness pre- and post-corrected assemblies for PsyTLP2, similar to the Psy508 results in Table 2. Set 1 is the primary Pseudomonas reference set of 11 equidistant genomes and Set 2 is comprised of 170 Pseudomonas genomes within Phylogroup 2. Using the BUSCO Pseudomonades database, 782 universal orthologs was classified as either complete, duplicated, fragmented, or missing.

| **Organism** | **Assembly Type** | | | **Correction** | **Reference set** |  | **Status of universal single orthologs** | | | |
| --- | --- | --- | --- | --- | --- | --- | --- | --- | --- | --- |
|  |  | | |  |  | *Total* | *Complete* | *Duplicated* | *Fragmented* | *Missing* |
|  | |  |  | |  |  |  |  |  |  |
| ***Pseudomonas syringae* TLP2** | Draft | | | - | - | 782 | 663 | 0 | 76 | 43 |
|  | Corrected | | | Kastor | Set 1 | 782 | 779 | 0 | 1 | 2 |
|  | Corrected | | | Kastor x2 | Set 2 | 782 | 779 | 0 | 1 | 2 |
|  | |  |  | |  |  |  |  |  |  |
| ***Pseudomonas syringae* TLP2 (DFM)** | Draft | | | - | - | 782 | 762 | 0 | 15 | 5 |
|  | Corrected | | | Kastor | Set 1 | 782 | 779 | 0 | 2 | 1 |
|  | Corrected | | | Kastor x2 | Set 2 | 782 | 780 | 0 | 1 | 1 |
|  |  | | |  |  |  |  |  |  |  |

1 –For testing of more recent assembly pipeline, dataset was re-basecalled using dorado, and assembled using a standard Flye and Medaka pipeline

Table S3. Number and types of errors detected by Kastor for PsyTLP2 (Pseudomonas Reference Set 1) and the impact of error correction on PGAP annotation.

|  | **PsyTLP2 assembly** | |
| --- | --- | --- |
| **Assembly Type** | Draft | Corrected |
| **Correction** | - | Kastor |
| **Reference set** | - | Set 1 |
| **Detected Errors** |  |  |
| Total | - | 3464 |
| Substitutions | - | 1279 |
| Deletions | - | 1413 |
| Insertions | - | 772 |
| **Indel Error Locations** |  |  |
| Within gene | - | 1487 |
| Intergenic | - | 697 |
| **Number of genes** |  |  |
| Complete | 3717 | 4758 (+1072/-24) |
| Frameshifted | 1202 | 173 (+6/-1034) |
| Incomplete | 130 | 97 (+13/-46) |

Table S5**.** Reference genome nucleotide distance chart for Pseudomonas Reference Set 1. Reference *P. syringae* genomes are labeled by strain name. Top-right values represent gapped identity while bottom-left values represent symmetrical identity

|  | 642 | UB0390 | B728a | B301D | SM | 10859 | HS191 | UMAF0158 | ICMP4048 | ISPaVe013 | ISPaVe037 |
| --- | --- | --- | --- | --- | --- | --- | --- | --- | --- | --- | --- |
| 642 | - | 98.73 | 94.35 | 94.28 | 94.42 | 94.5 | 94.34 | 94.04 | 94.11 | 94.73 | 94.61 |
| UB0390 | 88.85 | - | 94.35 | 94.35 | 94.56 | 94.59 | 94.45 | 94.13 | 94.16 | 94.73 | 94.64 |
| B728a | 78.67 | 79.12 | - | 98.62 | 94.94 | 95.03 | 94.92 | 94.44 | 94.62 | 95.44 | 95.435 |
| B301D | 78.64 | 79.12 | 90.94 | - | 94.92 | 95.02 | 94.86 | 94.45 | 94.66 | 95.41 | 95.45 |
| SM | 79.09 | 79.11 | 83.22 | 82.27 | - | 98.04 | 97.08 | 94.48 | 94.53 | 96.48 | 96.43 |
| 10859 | 80.01 | 80.09 | 84.52 | 84.25 | 86.8 | - | 97.18 | 94.58 | 94.76 | 96.63 | 96.56 |
| HS191 | 79.01 | 79.02 | 84.12 | 83.35 | 86.011 | 87.6 | - | 95.87 | 95.84 | 96.1 | 96.07 |
| UMAF0158 | 80.57 | 80.03 | 82.23 | 82.5 | 81.89 | 83.52 | 86.06 | - | 98.67 | 95.15 | 95.14 |
| ICMP4048 | 76.74 | 77.71 | 77.69 | 78.06 | 78.44 | 78.56 | 80.89 | 87.02 | - | 95.11 | 95.11 |
| ISPaVe013 | 76.71 | 77.84 | 80.6 | 79.59 | 80.41 | 80.93 | 79.92 | 79.85 | 77.68 | - | 98.16 |
| ISPaVe037 | 77.66 | 78.99 | 80.35 | 95.45 | 80.82 | 82.75 | 80.01 | 81.45 | 79.53 | 87.35 | - |

Table S6**.** Kastor computational resource use for error detection

| **Organism** | **Reference set^1^** | **Runtime^2^** | **Memory use (Gb)^2^** |
| --- | --- | --- | --- |
| ***Pseudomonas syringae* 508** | Set 1 | 0:05:30 | 5.45 |
|  | Set 2 | 0:15:30 | 7.27 |
|  |  |  |  |
| ***Pseudomonas syringae* TLP2** | Set 1 | 0:04:41 | 5.31 |
|  | Set 2 | 0:14:27 | 7.07 |
|  |  |  |  |
| ***Citrobacter koseri* MINF_9D** | Genus | 0:03:32 | 4.53 |
|  | Species | 0:03:49 | 4.31 |
|  |  |  |  |
| ***Mycobacterium tuberculosis*** | Species | 0:03:02 | 3.91 |
|  |  |  |  |
| ***Plasmodium falciparum* W2** | Species | 0:30:01 | 19.03 |
|  |  |  |  |

1 – See Supplementary File S4 for Reference set composition

2 – Computational resource use for Kastor as evaluated using native linux /usr/bin/time software. Runtime indicates wall clock time elapsed, and memory usage is reported as the maximum resident set size during the full run. Run times do not include generation of input alignments.

Table S7. Influence of genome set on Kastor corrections using BUSCO to assess completeness of Pss508. Three genome sets (differing in number and diversity of reference genomes and targeting different taxonomic levels) were used with Kastor. Set 1 - 11 equidistant *P. syringae* genome set; set 2 – 170 *P. syringae* phylogroup 2 genome set; set 3 – 441 *P. syringae* large genome set.

| **Genome set** | | | | **Status of universal single orthologs** | | | |
| --- | --- | --- | --- | --- | --- | --- | --- |
|  | Set number |  | *Complete* | | *Duplicated* | *Fragmented* | *Missing* |
|  | - |  | 385  451 | | 0  0 | 54  0 | 13  1 |
|  | Set 1 |  |  |  |  |  |  |
|  | Set 2 |  | 448 | | 0 | 11 | 4 |
|  | Set 3 |  | 426 | | 0 | 18 | 7 |

Table S8**.** Optional Kastor approach**:** Number of corrections using two genome sets to maximize assembly coverage (differing in number and diversity of reference genomes and targeting different taxonomic levels) for maximizing errors detected by Kastor. Set one is the primary set in all cases. Set 1 – 11 equidistant *P. syringae* genome set; set 2 – 170 *P. syringae* phylogroup 2 genome set; set 3 – 441 *P. syringae* genome set.

|  | **Assembly** | | | |
| --- | --- | --- | --- | --- |
|  | **Pss508** | | **Psy642** | |
| **Two genome sets^1^** | Set 1 + Set 2 | Set 1 + Set 3 | Set 1 + Set 2 | Set 1 + Set 3 |
| **Detected Errors** |  |  |  |  |
| Total | 3153 | 3077 | 222 | 219 |
| Substitutions | 969 | 947 | - | - |
| Deletions | 1857 | 1816 | 136 | 136 |
| Insertions | 327 | 314 | 86 | 83 |
| **Indel Error Locations^2^** |  |  |  |  |
| Within gene | 1445 | 1410 | 22 | 24 |
| Intergenic | 739 | 720 | 200 | 195 |
| **Number of genes** |  |  |  |  |
| Complete | 4943 (+1017 / -27) | 4927 (+997 / -23) | 4982 (+10 / -9) | 4983 (+11 / -9) |
| Frameshifted | 131 (+10 / -978) | 148 (+9 / -960) | 20 (+1 / -6) | 19 (+1 / -7) |
| Incomplete | 106 (+11 / -39) | 107 (+11/ -38) | 213 (+6 / -2) | 215 (+8 / -2) |

1 – Combination of set used (Primary set + secondary set supplementing low depth regions only).

2 – Error locations were determined based on assembly annotations using NCBI’s PGAP.
